# Systematic Cognitive Impairment Screening in Hospitalized Older Patients

**DOI:** 10.1002/alz70860_101651

**Published:** 2025-12-23

**Authors:** Zaldy S Tan, Nabeel Qureshi, Nancy Sicotte, Cameron Escovedo, Andrew Hirsch, Afshin Afrashteh, Peggy B Miles, Pamela Roberts

**Affiliations:** ^1^ Cedars‐Sinai Medical Center, Los Angeles, CA, USA; ^2^ UCLA David Geffen School of Medicine, Los Angeles, CA, USA; ^3^ RAND Corporation, Santa Monica, CA, USA

## Abstract

**Background:**

Over 40% of patients age 70+ admitted to the hospital have Alzheimer's disease or related dementias (ADRD), with the actual number likely to be higher as detection rates of ADRD in hospital settings have been shown to be less than 50%. We implemented a comprehensive program to identify patients with cognitive impairment at the point of hospitalization in a large, academic medical center to assess differences between groups based on cognitive impairment status.

**Method:**

To identify patients with cognitive impairment at hospital admission, we: 1. Modified the electronic health record (EHR) system to flag patients with dementia diagnosis or on a medication for ADRD; 2. Screened for cognitive impairment in patients age ≥65 years using the 4AT screen; and 3. Deployed the AD8 dementia interview in patients with positive 4AT screen. The screening and positivity rate for dementia, were calculated then ran Poisson and logistic regressions assessing differences in length of stay (LOS), readmission, and previous inpatient utilization by cognitive impairment level.

**Result:**

Of the 11,479 patient encounters over a 16‐month period, 2,012 (17.5%) had an ADRD diagnosis or medication, and 1,633 had delirium or cognitive impairment (14.2%). Of those, 454 had positive dementia interview (27.8%) while 224 were not screened (13.7%). The screening process increased the number of encounters with dementia by 22.6% (454/2,012). Patients with ADRD diagnosis or who had a positive dementia screen were older (age 85+, aOR=2.07, 95% CI: 1.66‐2.58), non‐white race (aOR=1.38, 95%CI: 1.07‐1.79), admitted from a medical setting (aOR=1.32, CI:1.18‐1.44), and had at least one previous hospitalization or emergency department visit in the past 6 months (aOR=2.53‐3.02). Undiagnosed cognitive impairment was associated with longer LOS (IRR=1.28, 95% CI: 1.91‐1.38) and more previous hospitalizations (IRR=1.25, 95% CI: 1.16‐1.34). Diagnosis of ADRD was associated with higher 90‐day readmissions (aOR: 1.25, 95% CI: 1.07‐1.46) and more previous hospitalizations (IRR=1.50, 95% CI: 1.35‐1.66).

**Conclusion:**

A comprehensive cognitive impairment screening program identified 23% more encounters with likely dementia in patients over the age of 65. The systematic and early identification of hospitalized patients with cognitive impairment has the potential to improve patient outcomes, optimize healthcare utilization, and reduce costs of care.

